# The genome sequence of the flea beetle, *Crepidodera aurea* (Geoffrey, 1785)

**DOI:** 10.12688/wellcomeopenres.22454.1

**Published:** 2024-06-13

**Authors:** Mark G. Telfer, Hermione Blomfield-Smith, University of Oxford and Wytham Woods Genome Acquisition Lab, Darwin Tree of Life Barcoding collective, Wellcome Sanger Institute Tree of Life Management, Samples and Laboratory team, Wellcome Sanger Institute Scientific Operations: Sequencing Operations, Wellcome Sanger Institute Tree of Life Core Informatics team, Tree of Life Core Informatics collective, Darwin Tree of Life Consortium

**Affiliations:** 1 Independent researcher (entomological consultant), Ventnor, Isle of Wight, England, UK; 2 Wellcome Sanger Institute, Hinxton, England, UK

**Keywords:** Crepidodera aurea, flea beetle, genome sequence, chromosomal, Coleoptera

## Abstract

We present a genome assembly from an individual female *Crepidodera aurea* (flea beetle; Arthropoda; Insecta; Coleoptera; Chrysomelidae). The genome sequence is 509.0 megabases in span. Most of the assembly is scaffolded into 10 chromosomal pseudomolecules, including the X sex chromosome. The mitochondrial genome has also been assembled and is 18.69 kilobases in length. Gene annotation of this assembly on Ensembl identified 19,944 protein coding genes.

## Species taxonomy

Eukaryota; Opisthokonta; Metazoa; Eumetazoa; Bilateria; Protostomia; Ecdysozoa; Panarthropoda; Arthropoda; Mandibulata; Pancrustacea; Hexapoda; Insecta; Dicondylia; Pterygota; Neoptera; Endopterygota; Coleoptera; Polyphaga; Cucujiformia; Chrysomeloidea; Chrysomelidae; Galerucinae; Alticini; Crepidoderina; *Crepidodera*; *Crepidodera aurea*, (Geoffrey, 1785) (NCBI:txid346762).

## Background

*Crepidodera aurea,* Geoffrey, 1785, is a small iridescent flea beetle from the sub-family Alticini ( Hubble, 2017). The Alticini beetles are thought to be the largest and most hyperdiverse sub-family of the Chrysomelidae ‘leaf’ beetles ( Letsch & Beran, 2023). *C. aurea* is widespread across Europe and large parts of Asia but largely inhabits only a few plant hosts ( Urban, 2011).

Flea beetles are unique from the leaf beetles due to their unique morphology. As the name suggests, flea beetles are equipped with a specialist 'meta-femoral spring’ mechanism in their hind legs allowing them to jump ( Hubble, 2017; Letsch & Beran, 2023). Flea beetles are small bodied (0.5–18 mm) with huge colour variability, many also being shiny or metallic ( Aslan & Ünal, 2022).

Collectively, the flea beetles feed on a wide variety of foliage, bushes and trees and can cause catastrophic damage ( Aslan & Ünal, 2022), although *C. aurea* has not been reported as an economically impactful pest ( Urban, 2011). *C. aurea* is oligophagous, with host species predominantly in the genera *Populus* and *Salix* ( Urban, 2011) and can cause large amounts of damage to the young leaves of its hosts ( Ünal *et al.*, 2016; Urban, 2011).

Further study of *C. aurea* and the Alticini beetles could inform phylogenetic relationships and diversification events ( Letsch & Beran 2023), but also gaps in literature on the life cycle and host plant associations of *C. aurea* and potential harmfulness of the species ( Urban, 2011).

## Genome sequence report

**Figure 1.**
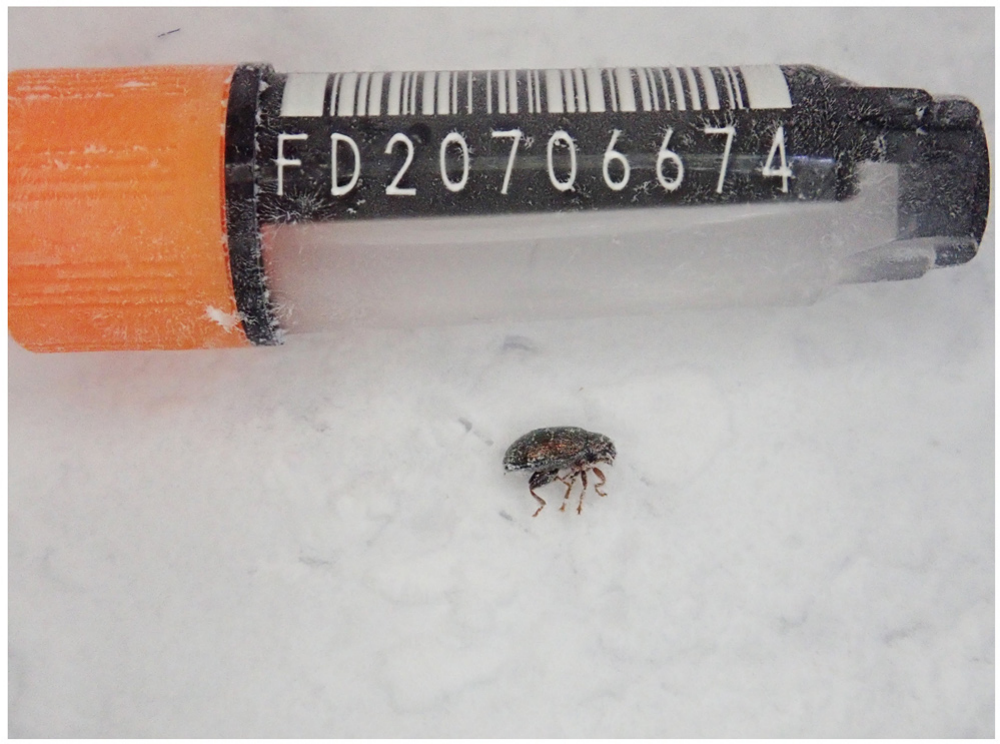
Photograph of the *Crepidodera aurea* (icCreAure2) specimen used for genome sequencing.

The genome was sequenced from one female *Crepidodera aurea* ( Figure 1) collected from Wytham Woods, Oxfordshire, UK (51.76, –1.34). A total of 44-fold coverage in Pacific Biosciences single-molecule HiFi long reads was generated. Primary assembly contigs were scaffolded with chromosome conformation Hi-C data. Manual assembly curation corrected 30 missing joins or mis-joins and removed 13 haplotypic duplications, reducing the assembly length by 1.35% and the scaffold number by 26.47%.

**Figure 2.**
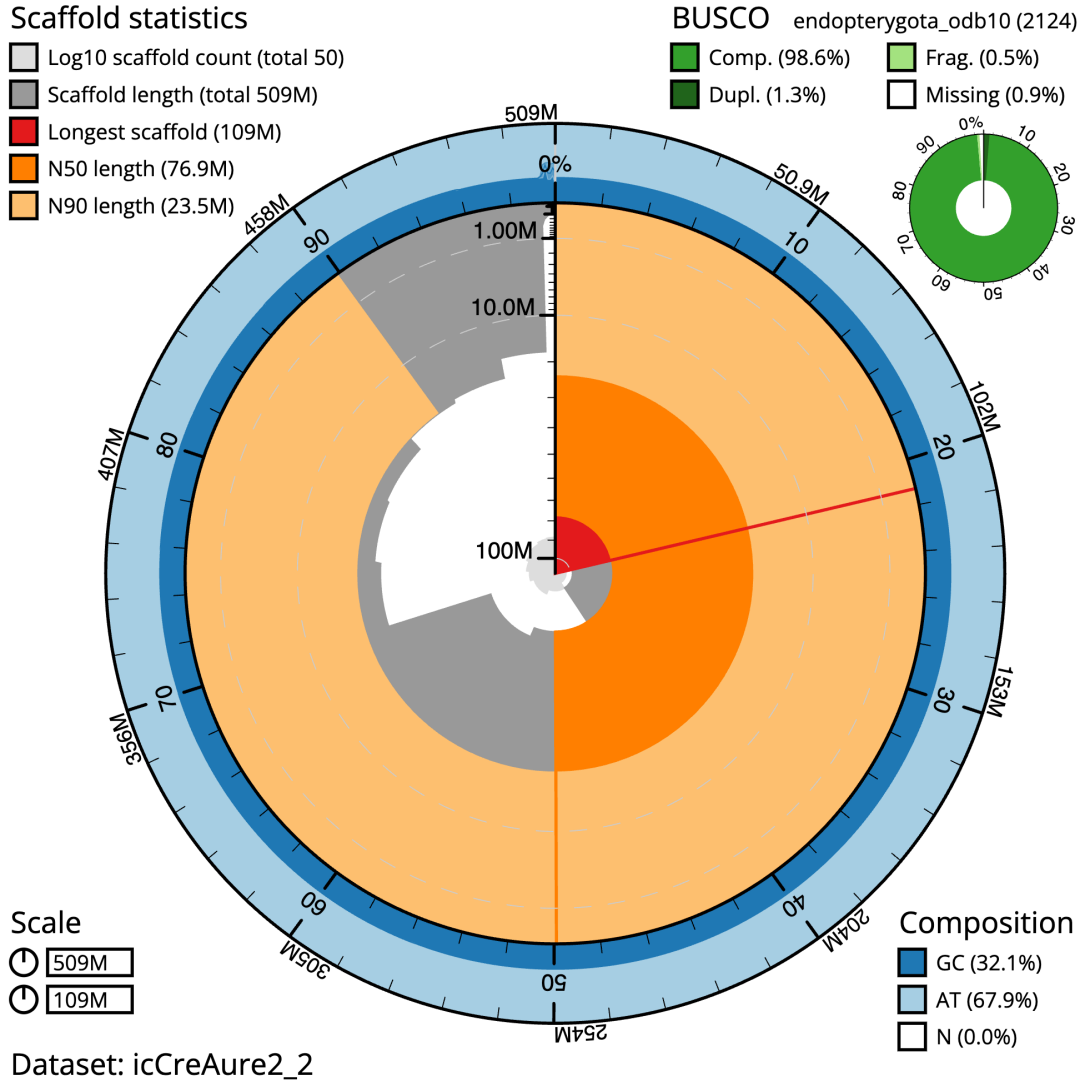
Genome assembly of *Crepidodera aurea*, icCreAure2.2: metrics. The BlobToolKit snail plot shows N50 metrics and BUSCO gene completeness. The main plot is divided into 1,000 size-ordered bins around the circumference with each bin representing 0.1% of the 508,975,589 bp assembly. The distribution of scaffold lengths is shown in dark grey with the plot radius scaled to the longest scaffold present in the assembly (108,592,676 bp, shown in red). Orange and pale-orange arcs show the N50 and N90 scaffold lengths (76,910,125 and 23,494,759 bp), respectively. The pale grey spiral shows the cumulative scaffold count on a log scale with white scale lines showing successive orders of magnitude. The blue and pale-blue area around the outside of the plot shows the distribution of GC, AT and N percentages in the same bins as the inner plot. A summary of complete, fragmented, duplicated and missing BUSCO genes in the endopterygota_odb10 set is shown in the top right. An interactive version of this figure is available at https://blobtoolkit.genomehubs.org/view/Crepidodera%20aurea/dataset/icCreAure2_2/snail.

**Figure 3.**
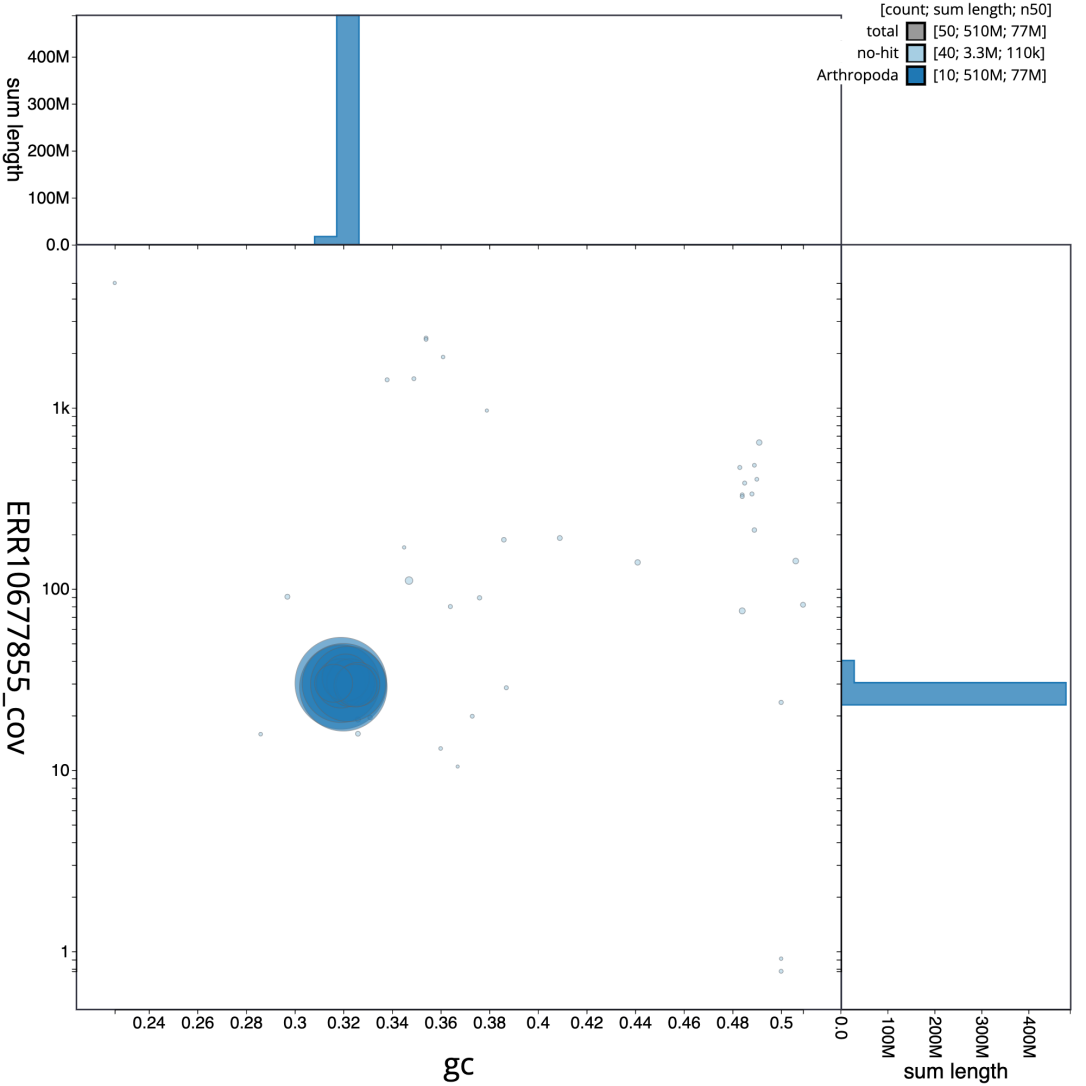
Genome assembly of *Crepidodera aurea*, icCreAure2.2: BlobToolKit GC-coverage plot. Sequences are coloured by phylum. Circles are sized in proportion to sequence length. Histograms show the distribution of sequence length sum along each axis. An interactive version of this figure is available at https://blobtoolkit.genomehubs.org/view/Crepidodera%20aurea/dataset/icCreAure2_2/blob.

**Figure 4.**
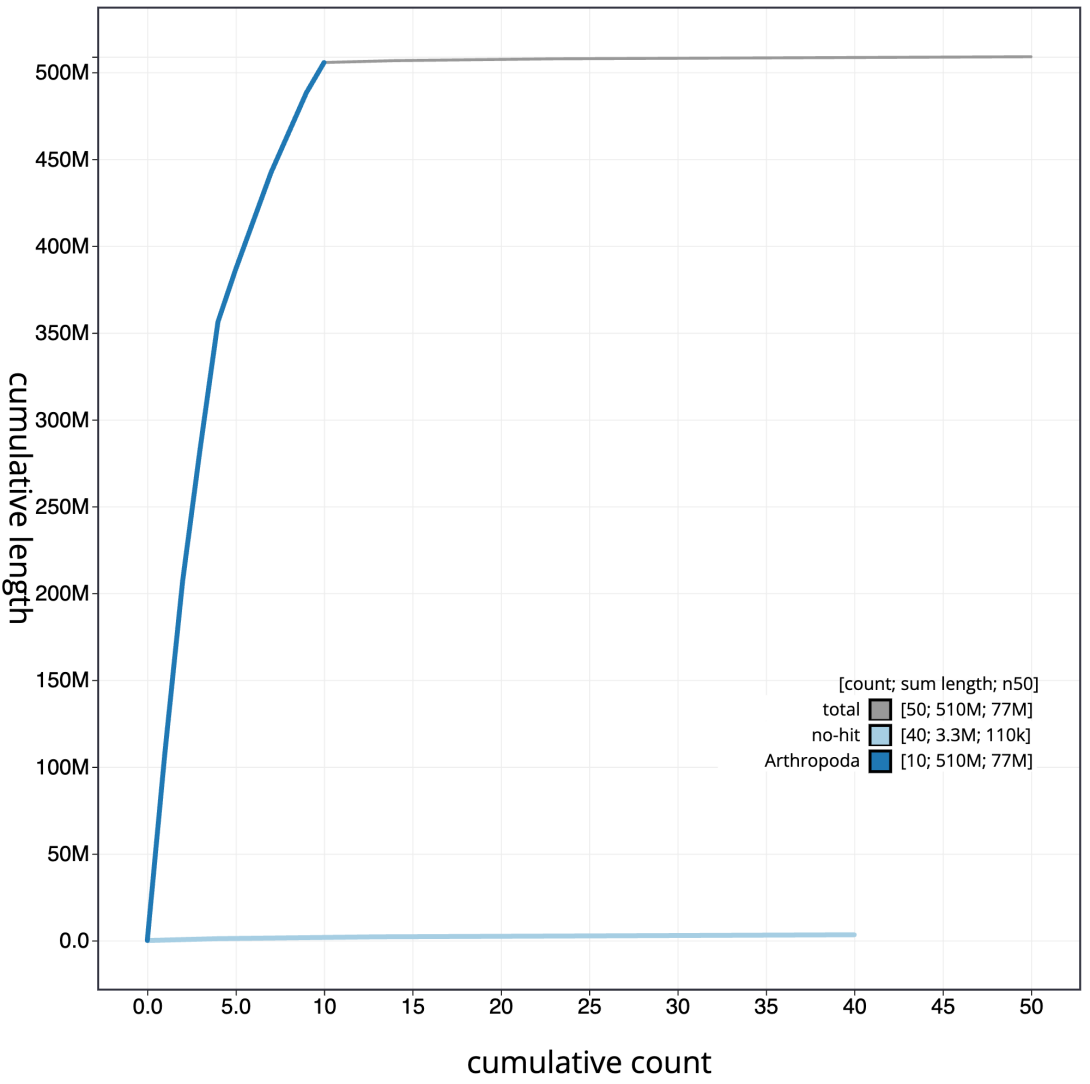
Genome assembly of *Crepidodera aurea*, icCreAure2.2: BlobToolKit cumulative sequence plot. The grey line shows cumulative length for all sequences. Coloured lines show cumulative lengths of sequences assigned to each phylum using the buscogenes taxrule. An interactive version of this figure is available at https://blobtoolkit.genomehubs.org/view/Crepidodera%20aurea/dataset/icCreAure2_2/cumulative.

**Figure 5.**
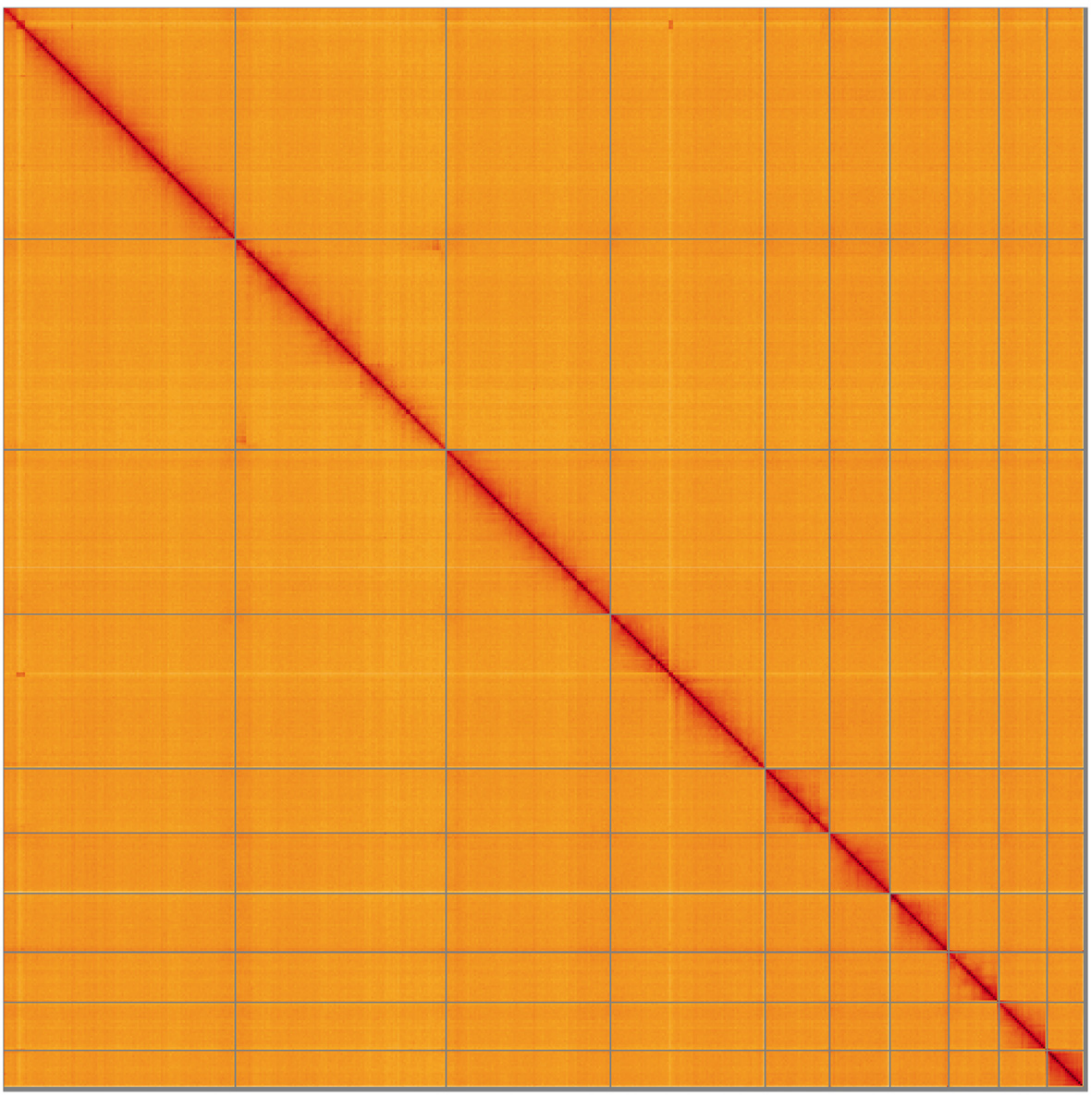
Genome assembly of *Crepidodera aurea*, icCreAure2.2: Hi-C contact map of the icCreAure2.2 assembly, visualised using HiGlass. Chromosomes are shown in order of size from left to right and top to bottom. An interactive version of this figure may be viewed at https://genome-note-higlass.tol.sanger.ac.uk/l/?d=Qv7pBuEGR1Wp0LljKwO9Bw.

The final assembly has a total length of 509.0 Mb in 49 sequence scaffolds with a scaffold N50 of 76.9 Mb ( Table 1). The snail plot in Figure 2 provides a summary of the assembly statistics, while the distribution of assembly scaffolds on GC proportion and coverage is shown in Figure 3. The cumulative assembly plot in Figure 4 shows curves for subsets of scaffolds assigned to different phyla. Most (99.58%) of the assembly sequence was assigned to 10 chromosomal-level scaffolds, representing 9 autosomes and the X sex chromosome. Chromosome-scale scaffolds confirmed by the Hi-C data are named in order of size ( Figure 5; Table 2). Chromosome X was assigned by synteny to *Coccinella septempunctata* (GCA_907165205.1) ( Crowley *et al.*, 2021). We observed a difference between the published karyotype ( Segarra & Petitpierre, 1988) and the chromosomal number based on the evidence for this assembly. While not fully phased, the assembly deposited is of one haplotype. Contigs corresponding to the second haplotype have also been deposited. The mitochondrial genome was also assembled and can be found as a contig within the multifasta file of the genome submission.

**Table 1.** Genome data for *Crepidodera aurea*, icCreAure2.2.

Project accession data		
Assembly identifier	icCreAure2.2	
Species	*Crepidodera aurea*	
Specimen	icCreAure2	
NCBI taxonomy ID	346762	
BioProject	PRJEB58254	
BioSample ID	Genome sequencing and Hi-C scaffolding: SAMEA10201276 RNA sequencing: SAMEA10201277	
Isolate information	icCreAure2: whole organism (PacBio HiFi and Illumina Hi-C sequencing) icCreAure3: whole organism (RNA sequencing)	
Assembly metrics *		*Benchmark*
Consensus quality (QV)	59.3	*≥ 50*
*k*-mer completeness	100.0%	*≥ 95%*
BUSCO **	C:98.6%[S:97.4%,D:1.2%], F:0.5%,M:0.9%,n:2,124	*C ≥ 95%*
Percentage of assembly mapped to chromosomes	99.58%	*≥ 95%*
Sex chromosomes	X	*localised homologous pairs*
Organelles	Mitochondrial genome: 18.69 kb	*complete single alleles*
Raw data accessions		
PacificBiosciences Sequel IIe	ERR10677855	
Hi-C Illumina	ERR10684083	
PolyA RNA-Seq Illumina	ERR12765122	
Genome assembly		
Assembly accession	GCA_949320105.2	
*Accession of alternate haplotype*	GCA_949319345.2	
Span (Mb)	509.0	
Number of contigs	293	
Contig N50 length (Mb)	4.4	
Number of scaffolds	49	
Scaffold N50 length (Mb)	76.9	
Longest scaffold (Mb)	108.66	
Genome annotation		
Number of protein-coding genes	19,944	
Number of gene transcripts	20,149	

* Assembly metric benchmarks are adapted from column VGP-2020 of “Table 1: Proposed standards and metrics for defining genome assembly quality” from Rhie *et al.* (2021). ** BUSCO scores based on the endopterygota_odb10 BUSCO set using version v5.4.3. C = complete [S = single copy, D = duplicated], F = fragmented, M = missing, n = number of orthologues in comparison. A full set of BUSCO scores is available at https://blobtoolkit.genomehubs.org/view/Crepidodera%20aurea/dataset/icCreAure2_2/busco.

**Table 2.** Chromosomal pseudomolecules in the genome assembly of *Crepidodera aurea*, icCreAure2.

INSDC accession	Chromosome	Length (Mb)	GC%
OX439472.1	1	108.59	32.0
OX439473.1	2	98.55	32.0
OX439474.1	3	76.91	32.0
OX439475.1	4	72.34	32.0
OX439476.1	5	30.14	32.0
OX439477.1	6	28.26	32.5
OX439479.2	7	27.49	32.0
OX439480.2	8	23.49	32.5
OX439481.2	9	22.47	32.5
OX439478.2	X	17.42	31.5
OX439482.1	MT	0.02	22.5

The estimated Quality Value (QV) of the final assembly is 59.3 with *k*-mer completeness of 100.0%, and the assembly has a BUSCO v5.3.2 completeness of 98.6% (single = 97.4%, duplicated = 1.2%), using the endopterygota_odb10 reference set ( *n* = 2,124).

Metadata for specimens, BOLD barcode results, spectra estimates, sequencing runs, contaminants and pre-curation assembly statistics are given at https://links.tol.sanger.ac.uk/species/346762.

## Genome annotation report

The *Crepidodera aurea* genome assembly (GCA_949320105.2) was annotated at the European Bioinformatics Institute (EBI) on Ensembl Rapid Release. The resulting annotation includes 20,129 transcribed mRNAs from 19,944 protein-coding genes ( Table 1; https://rapid.ensembl.org/Crepidodera_aurea_GCA_949320105.2/Info/Index).

## Methods

### Sample acquisition and nucleic acid extraction

*Crepidodera aurea* specimens were collected from Wytham Woods, Oxfordshire (biological vice-county Berkshire) (latitude 51.76, longitude –1.34) on 2021-05-25 by potting. The specimens were collected and identified by Mark Telfer (independent researcher), and then preserved on dry ice. A specimen with ID Ox001495 (ToLID icCreAure2) was used for PacBio DNA and Illumina Arima2 Hi-C sequencing, while a specimen with ID Ox001496 (ToLID icCreAure3) was used for RNA sequencing.

The workflow for high molecular weight (HMW) DNA extraction at the Wellcome Sanger Institute (WSI) Tree of Life Core Laboratory includes a sequence of core procedures: sample preparation; sample homogenisation, DNA extraction, fragmentation, and clean-up. The sample was prepared for extraction in the Tree of Life Core Laboratory: the icCreAure2 sample was weighed and dissected on dry ice ( Jay *et al.*, 2023), and tissue from the whole was homogenised using a PowerMasher II tissue disruptor ( Denton *et al.*, 2023a), setting aside tissue for Hi-C sequencing.

HMW DNA was extracted in the WSI Scientific Operations core using the Automated MagAttract v2 protocol ( Oatley *et al.*, 2023). The DNA was sheared into an average fragment size of 12–20 kb in a Megaruptor 3 system with speed setting 31 ( Bates *et al.*, 2023). Sheared DNA was purified by solid-phase reversible immobilisation ( Strickland *et al.*, 2023): in brief, the method employs a 1.8X ratio of AMPure PB beads to sample to eliminate shorter fragments and concentrate the DNA. The concentration of the sheared and purified DNA was assessed using a Nanodrop spectrophotometer and Qubit Fluorometer and Qubit dsDNA High Sensitivity Assay kit. Fragment size distribution was evaluated by running the sample on the FemtoPulse system.

RNA was extracted from whole organism tissue of icCreAure3 in the Tree of Life Laboratory at the WSI using the RNA Extraction: Automated MagMax™ *mir*Vana protocol ( do Amaral *et al.*, 2023). The RNA concentration was assessed using a Nanodrop spectrophotometer and a Qubit Fluorometer using the Qubit RNA Broad-Range Assay kit. Analysis of the integrity of the RNA was done using the Agilent RNA 6000 Pico Kit and Eukaryotic Total RNA assay.

Protocols developed by the WSI Tree of Life laboratory are publicly available on protocols.io ( Denton *et al.*, 2023b).

### Sequencing

Pacific Biosciences HiFi circular consensus DNA sequencing libraries were constructed according to the manufacturers’ instructions. Poly(A) RNA-Seq libraries were constructed using the NEB Ultra II RNA Library Prep kit. DNA and RNA sequencing was performed by the Scientific Operations core at the WSI on Pacific Biosciences Sequel IIe (HiFi) and Illumina NovaSeq X (RNA-Seq) instruments. Hi-C data were also generated from remaining tissue of icCreAure2 using the Arima v2 kit. The Hi-C sequencing was performed using paired-end sequencing with a read length of 150 bp on the Illumina NovaSeq 6000 instrument.

### Genome assembly and curation

Assembly was carried out with Hifiasm ( Cheng *et al.*, 2021) and haplotypic duplication was identified and removed with purge_dups ( Guan *et al.*, 2020). The assembly was then scaffolded with Hi-C data ( Rao *et al.*, 2014) using YaHS ( Zhou *et al.*, 2023). The assembly was checked for contamination and corrected using the gEVAL system ( Chow *et al.*, 2016) as described previously ( Howe *et al.*, 2021). Manual curation was performed using gEVAL, HiGlass ( Kerpedjiev *et al.*, 2018) and PretextView ( Harry, 2022). The mitochondrial genome was assembled using MitoHiFi ( Uliano-Silva *et al.*, 2023), which runs MitoFinder ( Allio *et al.*, 2020) or MITOS ( Bernt *et al.*, 2013) and uses these annotations to select the final mitochondrial contig and to ensure the general quality of the sequence.

### Final assembly evaluation

A Hi-C map for the final assembly was produced using bwa-mem2 ( Vasimuddin *et al.*, 2019) in the Cooler file format ( Abdennur & Mirny, 2020). To assess the assembly metrics, the *k*-mer completeness and QV consensus quality values were calculated in Merqury ( Rhie *et al.*, 2020). This work was done using Nextflow ( Di Tommaso *et al.*, 2017) DSL2 pipelines “sanger-tol/readmapping” ( Surana *et al.*, 2023a) and “sanger-tol/genomenote” ( Surana *et al.*, 2023b). The genome was analysed within the BlobToolKit environment ( Challis *et al.*, 2020) and BUSCO scores ( Manni *et al.*, 2021; Simão *et al.*, 2015) were calculated.

Table 3 contains a list of relevant software tool versions and sources.

**Table 3.** Software tools: versions and sources.

Software tool	Version	Source
BlobToolKit	4.1.7	https://github.com/blobtoolkit/blobtoolkit
BUSCO	5.3.2	https://gitlab.com/ezlab/busco
Hifiasm	0.16.1-r375	https://github.com/chhylp123/hifiasm
HiGlass	1.11.6	https://github.com/higlass/higlass
Merqury	MerquryFK	https://github.com/thegenemyers/MERQURY.FK
MitoHiFi	2	https://github.com/marcelauliano/MitoHiFi
PretextView	0.2	https://github.com/wtsi-hpag/PretextView
purge_dups	1.2.3	https://github.com/dfguan/purge_dups
sanger-tol/genomenote	v1.0	https://github.com/sanger-tol/genomenote
sanger-tol/readmapping	1.1.0	https://github.com/sanger-tol/readmapping/tree/1.1.0
YaHS	yahs-1.1.91eebc2	https://github.com/c-zhou/yahs

### Genome annotation

The BRAKER2 pipeline ( Brůna *et al.*, 2021) was used in the default protein mode to generate annotation for the *Crepidodera aurea* assembly (GCA_949320105.2) in Ensembl Rapid Release at the EBI.

### Wellcome Sanger Institute – Legal and Governance

The materials that have contributed to this genome note have been supplied by a Darwin Tree of Life Partner. The submission of materials by a Darwin Tree of Life Partner is subject to the **‘Darwin Tree of Life Project Sampling Code of Practice’**, which can be found in full on the Darwin Tree of Life website here. By agreeing with and signing up to the Sampling Code of Practice, the Darwin Tree of Life Partner agrees they will meet the legal and ethical requirements and standards set out within this document in respect of all samples acquired for, and supplied to, the Darwin Tree of Life Project.

Further, the Wellcome Sanger Institute employs a process whereby due diligence is carried out proportionate to the nature of the materials themselves, and the circumstances under which they have been/are to be collected and provided for use. The purpose of this is to address and mitigate any potential legal and/or ethical implications of receipt and use of the materials as part of the research project, and to ensure that in doing so we align with best practice wherever possible. The overarching areas of consideration are:

•     Ethical review of provenance and sourcing of the material

•     Legality of collection, transfer and use (national and international)

Each transfer of samples is further undertaken according to a Research Collaboration Agreement or Material Transfer Agreement entered into by the Darwin Tree of Life Partner, Genome Research Limited (operating as the Wellcome Sanger Institute), and in some circumstances other Darwin Tree of Life collaborators.

## Data Availability

European Nucleotide Archive: *Crepidodera aurea*. Accession number PRJEB58254; https://identifiers.org/ena.embl/PRJEB58254 ( Wellcome Sanger Institute, 2023). The genome sequence is released openly for reuse. The *Crepidodera aurea* genome sequencing initiative is part of the Darwin Tree of Life (DToL) project. All raw sequence data and the assembly have been deposited in INSDC databases. Raw data and assembly accession identifiers are reported in Table 1.

## Author information

Members of the University of Oxford and Wytham Woods Genome Acquisition Lab are listed here: https://doi.org/10.5281/zenodo.7125292.

Members of the Darwin Tree of Life Barcoding collective are listed here: https://doi.org/10.5281/zenodo.4893703.

Members of the Wellcome Sanger Institute Tree of Life Management, Samples and Laboratory team are listed here: https://doi.org/10.5281/zenodo.10066175.

Members of Wellcome Sanger Institute Scientific Operations: Sequencing Operations are listed here: https://doi.org/10.5281/zenodo.10043364.

Members of the Wellcome Sanger Institute Tree of Life Core Informatics team are listed here: https://doi.org/10.5281/zenodo.10066637.

Members of the Tree of Life Core Informatics collective are listed here: https://doi.org/10.5281/zenodo.5013541.

Members of the Darwin Tree of Life Consortium are listed here: https://doi.org/10.5281/zenodo.4783558.
