# Supplementary material for: The genome sequence of the flea beetle, Crepidodera aurea (Geoffrey, 1785)
Source: Wellcome Open Res. 2024 Jun 13;9:318. [Version 1] doi: 10.12688/wellcomeopenres.22454.1 (PMC11422754; doi:10.12688/wellcomeopenres.22454.1)
Supplement: Peer review history

# Reviewer report: Reviewer response for version 1

**DOI:** 10.21956/wellcomeopenres.24741.r97911

**Published:** 2024-09-24

**Authors:** Bryan Brunet

**Affiliations:** 1 Agriculture and Agri-Food Canada , Ottawa Research and Development Centre, Ottawa, Ontario, Canada

The data note entitled “The genome sequence of the flea beetle, *Crepidodera aurea* (Geoffrey, 1785)" describes efforts to sequence, assemble and annotate the genome of the species named therein, a species distributed throughout Europe but of little concern economically. The authors, Telfer and Blomfield-Smith, have utilized robust and publicly available workflows published by the Wellcome Sanger Institute Tree of Life laboratory to develop the genome assembly using a combination of long read, chromosome conformation capture and rna-seq sequence datasets. The assembly appears to be highly contiguous and complete with excellent genome assembly quality metrics. It also includes the complete mitochondrial genome. With the minor revisions identified below, I understand the work to be of scientific interest and soundness and therefore approve of its indexing.

**Minor revisions:**

**Background**

- *Crepidodera aurea* (Geoffrey, 1785) – taxonomic reference is incorrect as the authority should be in parentheses.
- Alticini is a tribe not a sub-family.
- Don’t abbreviate genus at the start of sentences
- Do the authors mean “unique among leaf beetles”? They’ve already stated that flea beetles belong to the leaf beetle family, Chrysomelidae.

**Genome sequence report**

- Figure 1 – This photograph is not useful diagnostically if that is its intent. In its current state, I cannot see a use for it other then to show that a specimen exists which is not very useful. If the intent is to provide some visual representation of the species, then specimen should be properly prepared on a pin or point according to standard practice and photographed with sufficient magnification so as to maximize specimen/species visibility and recognition.
- Where have the physical voucher(s) been retained?
- Figure 3 – Incomplete caption. Please describe the axes of the main cov by gc plot.
- Figure 4 – Please describe what is meant by “buscogenes taxrule”. This wasn’t obvious to me and I’ve used Blobtools for similar purposes.
- Please show synteny data and results from comparisons to *Coccinella septempunctata.*
- Please elaborate on why there are differences from the published karyotype.

**Methods: Genome assembly and curation**

- Please describe in greater detail the manual curation steps taken to correct the assembly on the basis of the HiC contact maps (eg. identifying scaffolds/chromosomes affected by missing/mis-joins as well as duplications). Simply stating the number would not allow for reproducibility of manual curation steps.

Are sufficient details of methods and materials provided to allow replication by others?

Partly

Is the rationale for creating the dataset(s) clearly described?

Yes

Are the datasets clearly presented in a useable and accessible format?

Yes

Are the protocols appropriate and is the work technically sound?

Yes

Reviewer Expertise:

My expertise is in aphid molecular systematics, and I've been involved in several genome assembly projects of various insects.

I confirm that I have read this submission and believe that I have an appropriate level of expertise to confirm that it is of an acceptable scientific standard.

# Reviewer report: Reviewer response for version 1

**DOI:** 10.21956/wellcomeopenres.24741.r97909

**Published:** 2024-09-16

**Authors:** Doga Cedden

**Affiliations:** 1 University of Göttingen, Göttingen, Germany

The data note by Telfer et al. presents a high-quality assembly from the flea beetle *Crepidodera aurea. *The authors provide sufficient background information about the insect, and the method is appropriate. The authors provide a sufficient quality assessment of the genome assembly. The genome assembly is publicly available, as described in the data note. I do not have any further suggestions.

Are sufficient details of methods and materials provided to allow replication by others?

Yes

Is the rationale for creating the dataset(s) clearly described?

Yes

Are the datasets clearly presented in a useable and accessible format?

Yes

Are the protocols appropriate and is the work technically sound?

Yes

Reviewer Expertise:

I work on beetle pests using RNAi and transcriptomic methods.

I confirm that I have read this submission and believe that I have an appropriate level of expertise to confirm that it is of an acceptable scientific standard.
